# Intersecting sex-related inequalities in self-reported testing for and prevalence of Non-Communicable Disease (NCD) risk factors in Kerala

**DOI:** 10.1186/s12889-022-12956-w

**Published:** 2022-03-19

**Authors:** Jyotsna Negi, Hari Sankar D, Arun B. Nair, Devaki Nambiar

**Affiliations:** 1 Independent Consultant, 62 Stratford Road, Kensington, CA 94707 USA; 2 The George Institute for Global Health, New Delhi, India; 3 Health Systems Research India Initiative, Thiruvananthapuram, Kerala India; 4 Faculty of Medicine, University of New South Wales, Sydney, Australia; 5 Prasanna School of Public Health, Manipal Academy of Higher Education, Manipal, India

**Keywords:** India, Kerala, NCDs, Inequalities, Screening, Hypertension, Diabetes

## Abstract

**Background:**

Non-Communicable Diseases (NCDs) are among India’s top burdens, particularly in states like Kerala, which is at an advanced stage of the epidemiological transition. Evidence in India points towards intersectional inequalities in risk factors of NCDs and testing, both of which are understudied in Kerala. We estimated the self-reported testing and prevalence of key NCD risk factors—blood pressure (BP) and blood glucose (BG) comparing Kerala men and women across educational, wealth, religion, as well as caste and tribal status subgroups.

**Method:**

A multistage random sample survey of 3398 women and 2982 men aged 30 years and over was administered in 4 districts of Kerala from July to October 2019. Descriptive analysis for men and women was undertaken using survey weights. Slope index of Inequality and Relative Concentration Index for wealth and education related inequalities, and, Weighted Mean Difference from Mean and Index of Disparity for caste and tribal status, as well as religion related inequalities were calculated using World Health Organisation’s Health Equity Assessment Toolkit Plus and Stata 12.

**Results:**

A significantly higher proportion of women reported BP and BG testing by medical personnel in the previous year than men (BP Testing among Women (BPT_w_): 90.3% vs BP Testing among Men (BPT_M_):80.8%, BG Testing among Women (BGT_w_): 86.2% vs BG Testing among Women (BGT_M_):78.3%). Among those tested, more women (11.2%) than men (7.9%) reported High Blood Pressure (HBP) but not High Blood Glucose (HBG). Testing for BP was concentrated among less-educated women while BG testing was concentrated among both less educated women and men. HBP and HBG were concentrated among less educated and wealthier groups. Although sex differences were insignificant across caste and tribal status and religion subgroups, magnitude of inequalities was high for HBP and HBG.

**Conclusion:**

Distinct patterns of sex inequalities were present in self-reported testing and prevalence of NCD risk factors in Kerala. Education and wealth seem to be associated with testing while prevalence appeared to vary by religious groups. Given the low rates of illiteracy, it is encouraging but maybe a data artefact that a small population of less-educated persons was getting tested; however, exclusion of poor groups and inequalities by other dimensions raise concerns. Further exploration is needed to understand underlying mechanisms of these inequalities to ensure we leave no one behind.

**Supplementary Information:**

The online version contains supplementary material available at 10.1186/s12889-022-12956-w.

## Background

The Sustainable Development Goals (SDGs), launched in 2015, identified Non-Communicable Diseases (NCDs) as a major threat to attain sustainable development and set a global target to reduce premature mortality by a third over the next 15 years [1]. The southern Indian state of Kerala ranked at the top of the country’s Sustainable Development Goal India Index in 2019 [2], besting other states for indicators like life expectancy, mortality and death rates. However, the morbidity levels in the state are comparatively much higher when compared with other Indian states, driven largely by NCDs burden [3–5]. A recent study reported that about 61% of households ailing from NCDs faced catastrophic health expenditures in Kerala, and in fact, absolute impoverishment for households due to NCDs burden in Kerala was the highest of any state in the country (20.7%) [6].

In light of this, the Government of Kerala has introduced state specific SDGs which include a target to reduce the prevalence of high blood pressure (HBP) by 30–40% and high blood glucose (HBG) by 18–20% among above 30 years of age group [7]. To achieve these state specific goals, Kerala has launched Aardram mission with an objective to transform public health systems placing special emphasis on increasing the scope and quality of primary care services. As a part of this mission, beginning in 2017, a number of the state’s Primary Health Centres (PHC) were upgraded to Family Health Centres (FHC) by increasing staff, training, infrastructure and working hours. Additionally, the state has revised NCDs guidelines to provide opportunistic screening for those aged above 30 for diabetes and above 18 years for hypertension [8].

Given these reforms, there was a need to identify population coverage at this early stage as a kind of baseline. As the FHC program was being rolled out, there was already evidence suggestive of inequalities: Kerala’s Economic Review 2018 reported sex differences in the prevalence of diabetes where 27% of adult males and 19% of adult females had diabetes [7].

Evidence from literature suggests variations in the prevalence of self-reported NCDs between men and women across countries [9–11], in India and Kerala [3, 12–27]. Sex, education, and income are associated with the prevalence of NCDs and their risk factors, leading to catastrophic disease burden among vulnerable populations [3, 9–14, 17–22, 25, 28]. A study published in 2012 found that Scheduled Caste (SC) status, Scheduled Tribe (ST) status, higher education, higher wealth status and increasing age were associated with a higher prevalence of diabetes [10]. Studies in Kerala have shown that persons below poverty line group in rural areas were less likely to have diabetes, hypertension or dyslipidaemia when compared with those above the poverty line [21], and also that sex differences exist in self-reported diabetes and are more prevalent among higher socio economic groups [20]. While self-reported prevalence of cardiometabolic risk factors has its limitations for drawing inferences on NCD outcomes [23], from a health systems perspective, this indicator can serve as a proxy of initial program outreach.

It is also the case that few of the aforementioned studies using clinical measurement were able (or powered) to assess NCD testing and prevalence of risk factors in intersectional population groups (i.e. men and women across socio-economic, social, and other groups). Intersectional analyses have revealed important insights into groups that are uniquely affected by morbidity as well as the reach and programs of the health system [10, 14, 18, 20–24]. Filling this gap, we sought to examine inequalities in testing and self-reported prevalence of HBP and HBG by education, wealth, caste and tribes and religion separately for men and women in Kerala. We drew upon a health systems survey undertaken to monitor the performance of Kerala’s Aardram health reform initiatives as a part of a larger implementation research study assessing equity in Universal Health Coverage reforms in the state [29, 30].

## Materials and methods

### Study design and setting

A multistage, random sample survey was undertaken from July to October 2019, powered to assess sex differences in self-reported testing of Blood Pressure (BP)/Blood Glucose (BG) and prevalence for HBP/HBG in the previous year among those aged 30 or older in Kerala. The state’s 14 districts were categorised into four groups by deriving an index using principal component analysis, a dimension-reduction tool on health burden and systems performance indicators from the National Family Health Survey (NFHS) Round 4 (2015–16) [31]. One district was chosen from each of the four groups randomly and two facilities per district were randomly selected. The sampling frame was reviewed and concurrence was received from state officials. Further details of the study design and sample size estimation methodology are presented in Additional file 4.

### Sampling

The required sample size for this survey was calculated to allow an estimation of sex differences for BP testing in the previous year within a ± 8% margin of error with 95% confidence probability, in consultation with a senior biostatistician. The proportion of eligible males and females of age greater than or equal to 30 years whose BP was measured in the previous year was obtained from data provided by NCD program of the Kerala Health Department. A conservative design effect of 2, was considered for the sample of our study. Health facility catchment areas were grouped by wards, the Primary Sampling Unit (PSU). All wards were selected in the five facilities where number of wards were less than equal to 20. In the remaining three facilities where the number of wards were more than 20, wards were stratified into 5 or 6 strata on the basis of population and four wards were selected randomly from these stratum such that total 20 wards are selected in each facility. Since most of the selected wards constituted more than 300 households per ward (total number of households in each ward in the previous year were obtained using estimates from Kerala’s electronic health information or E health platform), there was a requirement to create ward segmentations. With the help of ward members (elected representatives of the ward)/Junior Health Inspectors and locals, the field team created outline maps of each PSU with roads, major landmarks, and households plotted on the map. All PSUs of more than 300 households were divided into 2 or 3 segments of roughly equal size (some variation in size was expected) and a number was assigned to each segment. Based on guidance from statisticians, we created, enumerated and then randomly selected 20 non-overlapping, artificial ward segments from each ward within a selected PHC/FHC.

**Fig. 1:**
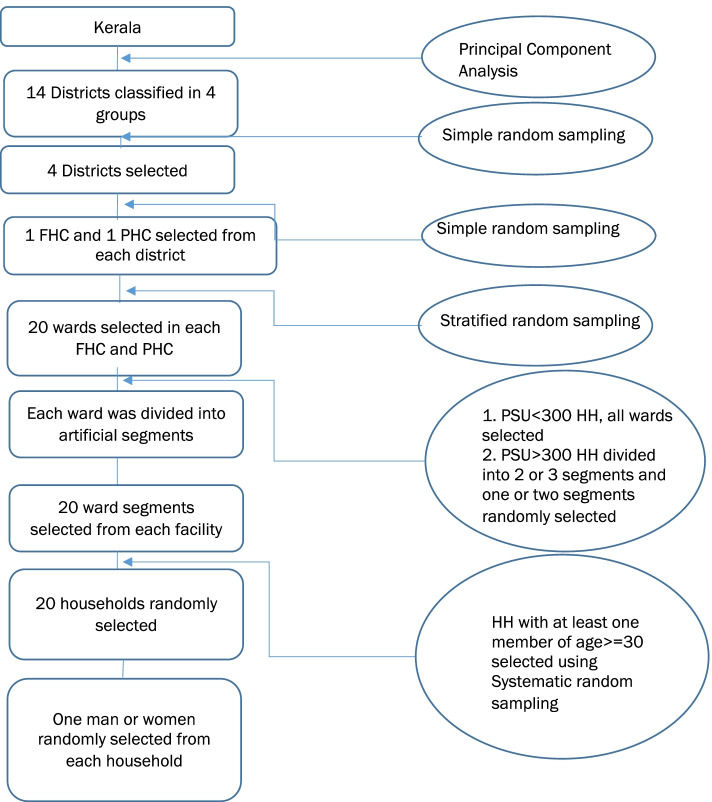
Sampling Design flow chart

For the purpose of selecting households, since no readily available sampling frame existed, 20 households were selected from each selected ward segment using systematic random sampling method. Households with at least one member in the age group 30 and above were eligible for selection. The definition of a household was similar to that used in NSSO 71st round on health: a group of persons normally living together and taking food from a common kitchen [32]. This included short-term stay-away (those whose total period of absence from the household was expected to be less than 6 months) but excluded temporary visitors and guests (expected total period of stay was less than 6 months). One person aged 30 or older was randomly selected from each household to get information on NCD testing and risk factors (HBP and HBG). Graphical representation of the sampling design is presented in the form of flow chart in Fig. 1.

### Field team recruitment and training

Data collection of the study was done by hiring local field staff (eight field investigators, two supervisors and one co-ordinator). The field team was given a five-day residential training in the local language (Malayalam) and a training manual. The investigators were familiarised with Kerala health system, services offered by health department, use of bilingual survey app for data collection and interview etiquettes. Mock drills were conducted among the group and field testing was done in a nearby ward of the training facility. Supervisors were also trained on creating outline maps and the ward segmentation procedure. During data collection, additional three field investigators were hired, oriented and given basic training by supervisors followed by ‘ride alongs’ with their colleagues to further orient themselves on data collection.

### Data collection

We conducted an interviewer administered survey using electronic tabs through a web-enabled structured questionnaire. The questionnaire was designed in Malayalam and English and field staff had the choice to select the language they were comfortable with. This questionnaire had 12 modules and captured information on household and individual socio-demographics, testing of BP/BG, and lifestyle attributes such as smoking, drinking, and physical activity. Additionally, information on hospitalisation, outpatient and chronic expenses during the year prior to the survey was gathered, as well as data on satisfaction with FHC/PHC visits, and awareness of services under the Aardram mission specifically requested by department officials was also collected.

Ethics approval of the study was received from the institutional ethics committee of George Institute for Global Health (Project Number 05/2019). All participants gave written informed consent before taking part in the study.

### Data processing

Real time data was collected through the tab and was automatically uploaded to a secure local server. Data exported from the server in excel format was cleaned, cross-checked and triangulated with multiple related questions by supervisors daily. Daily forms were provided to each investigator and supervisor to keep a quality check of the procured data. In case of discrepancies, data was re-entered in the tab by supervisors. Similar data collection procedure was used across all sites and for all respondents. The data collected as part of the study was stored in a secure server with access only to the research team members adhering to relevant national, state, and institutional data storage protocols.

### Data analysis

Descriptive analysis (mean, standard error (se) and 95% confidence interval (CIs)) was carried out for men and women separately for four self-reported indicators using survey weights.^1^: (i) BP testing (BPT) coverage, (ii) BG testing (BGT) coverage, (iii) prevalence of HBP, and (iv) prevalence of HBG. Chi square tests were used to determine significant sex differences for these four indicators. In addition, inequality analyses were carried out with summary measures using the World Health Organisation’s Health Equity Assessment Toolkit (HEAT) Plus [33] and Stata 12 [34].

The main outcome variables were self-reported testing for BP, testing for BG and prevalence of self-reported HBP and self-reported prevalence of HBG in men and women of age greater than or equal to 30 years in the previous year. Specifically, participants were asked “When was the last time you or doctor/nurse/other medical personnel measured your BP/BG?”. They were considered to have been “tested” if they responded with “during last six months or last year”. Participants were considered as reporting “high BP/BG levels” if they responded that a doctor/nurse/other medical personnel had told them their level was “higher than normal” during most recent BP/BG measurement in the previous year.

With regard to dimensions of inequality, sex was defined in categories: male, female and other sex. The sample for ‘other sex’ was too small to allow intersectional analysis and thus had to be excluded from further analysis. Level of education, wealth status, religion and caste were considered in the study as dimensions to assess inequalities in NCD testing and risk factors. Education was recoded into four categories from illiterate to higher secondary and above. We collected information on household assets, land holding, and nature of the dwellings, including type of toilets, water source, and source of cooking. Questions were also asked to report the possession of household assets such as radio, television, phone, electricity, fan, washing machine, microwave, car, scooter etc. Principal Component Analysis was performed to construct a wealth index using these asset related questions and wealth quintiles were constructed using this index. Assets owned by fewer than 5% and greater than 95% were not included in the analysis for obtaining wealth quintiles. Religion was recoded into four categories- Hindu, Muslim, Christian, and others. Caste and Tribal status was also recoded into four categories: ‘Scheduled Tribe,’ ‘Scheduled Caste’, ‘Other Backward Classes (OBC)’ and ‘General’ following the convention commonly used in Indian household surveys [31, 35]. An additional category ‘prefer not to say’ was added to caste and religion related variables for descriptive analysis; for inequality analyses, these participants were excluded.^2^

We assessed inequalities for men and women for each of the four selected indicators, relying on both absolute and relative, and simple and complex summary measures. Additional files include data on disaggregated analyses as well as simple measures of inequality (see Additional file 1); here we report main findings from absolute and relative complex measures. Complex summary measures are more representative of population subgroups than simple measures as the former draws data from all populations groups unlike the latter that takes into account only two population groups (e.g. least educated and most educated) [36]. While absolute measures provide absolute health difference between subgroups and retain the same unit of measure, relative measures provide the proportion of health differences between subgroups and are unitless [37]. The absolute and relative measures both can show different conclusions and have their own significance [37]. Therefore, both measures of inequality were reported in this study.

We computed summary measures of inequality and 95% Cis by education, wealth, caste, and religious group using Stata 12 [34] and the World Health Organisation’s HEAT plus [37]. We assessed the magnitude of inequality within both sex dimensions using an appropriate summary measure. For ordered dimensions-education and wealth, we used Slope Index of Inequality (SII) and Relative Concentration Index (RCI) whereas for non-ordered dimensions-caste and tribal status as well as religion, we used weighted Mean Difference from mean (MDM) and Weighted Index of Disparity (IDIS_W). SII is an absolute measure of inequality that uses a generalised linear regression model to calculate the predicted values of an indicator accounting for all population subgroups [33]. RCI is a relative measure that is measured by dividing the absolute concentration index (calculated by ranking whole population from less disadvantaged to most advantaged) to the setting average and multiplying by 100 [33]. MDM, a weighted absolute measure, shows the weighted mean of difference in each population subgroup from a reference subgroup [33]. IDIS_W is a relative measure that shows weighted mean difference between each population subgroup and the average of the population subgroups for the variable of interest [33]. More information about these measures is provided in Additional file 2. We also computed Cis to determine whether there were significant differences between sexes in magnitude of the same dimension of inequality.

## Results

Our data comprised a weighted sample of 3398 women and 2982 men (our overall survey response rate was 84.5%). Table 1 below presents demographics of the surveyed population. The mean age of the sample was 54.5 years, ranging from 30 to 99 years. The difference in education levels was significant between men and women in our sample (significantly fewer men than women were illiterate (*p* < 0.001)). The proportion of men who had completed ‘Higher Secondary and above’ was higher than women (W: 54.7% vs M: 61.6%). As expected, approximately 38% of the men and women belonged to the bottom two wealth quintiles. Most of the respondents belonged to Hindu religion (W: 65.1%, M: 65.6%) followed by Muslims and Christian. About two thirds (W:62.5% and M: 63.2%) belonged to the OBC category among both men and women. It must be noted that while other proportions approximate average levels in NFHS 5 held around the same time, our sample of OBC population (65.7%) is higher than that reported for Kerala overall (50%) [38].

**Table 1:** Participant socio-demographics

Demographics	Proportions % (n)^a^		Pearson Chi-square *p* value
	Women (*N* = 3398)	Men (*N* = 2982)	
**Age**			
30–44	26.7 (909)	25.4 (757)	0.436
45–59	36.0 (1224)	37.8 (1126)	
60+	37.2 (1265)	36.9 (1100)	
**Level of Education**			**0.004**
Illiterate	7.5 (254)	3.1 (93)	
Primary	2.12 (72)	1.13 (34)	
Secondary	35.7 (1214)	34.1 (1018)	
Higher Secondary and above	54.7 (1857)	61.6 (1837)	
**Wealth**			0.101
Poorest (Quintile 1)	18.4 (502)	16.8 (624)	
Poor (Quintile 2)	20.0 (629)	21.1 (680)	
Middle (Quintile 3)	23.2 (704)	23.6 (789)	
Richer (Quintile 4)	17.5 (520)	17.5 (595)	
Richest (Quintile 5)	20.9 (627)	21.0 (710)	
**Religion**			0.198
Hindu	65.1 (2210)	65.6 (1955)	
Muslim	18.7 (635)	17.6 (523)	
Christian	16.2 (549)	16.6 (498)	
Prefer not to say	0.1 (4)	0.2 (6)	
**Caste and tribal status**			0.602
Schedule Caste (SC)	7.4 (250)	7.3 (217)	
Tribal Status (ST)	2.2 (76)	1.9 (57)	
Other Backward Class (OBC)	62.5 (2123)	63.2 (1884)	
General	27.4 (929)	27 (806)	

Bold *p*-values indicate statistically significant sex differences. The sample also had ”other gender” which was excluded from the analysis due to insufficient sample size. Prefer not to say/Don’t know were excluded from inequity analysis

^a^n refers to numerator values. Due to rounding,-totals may not match

### Overall self-reported coverage of blood pressure testing (BPT), blood glucose testing (BGT), and self-reported prevalence of high blood pressure (HBP) and high blood glucose (HBG)

Proportions of men and women reporting BPT and BGT in the previous year respectively, along with the self-reported prevalence of HBP and HBG are presented in Table 2. BPT and self-reported prevalence of HBP were higher for women as compared to men (BPT_w_: 90.3% vs BPT_M_: 80.8%, *p* < 0.05 & HBP_w_: 11.2% vs HBP_M_: 7.9%, *p* < 0.05). The coverage of BGT was different for men and women but self-reported prevalence of HBG was not. Testing of BG was higher among women than men (BGT_w_: 86.2% vs BGT_M_: 78.3%, *p* < 0.05). Self-reported BPT, BGT, HBP and HBG self-reported prevalence disaggregated by each selected dimensions of inequality are reported in Additional file 1.

**Table 2:** Self-Reported Blood Pressure Testing (BPT), Blood Glucose Testing (BGT), and self-reported prevalence of High Blood Pressure (HBP) and High Blood Glucose (HBG)

	Women		Men	
	% (95%CI)	N	% (95%CI)	N
Proportion of those eligible (aged 30 years and over) reporting that their Blood Pressure was measured by doctor/nurse/other medical personnel in the previous year**	90.3 (89.1, 91.4)	3393	80.8 (78.5, 82.9)	2975
Of those whose Blood Pressure was measured in the previous year, proportion of individuals reporting High Blood Pressure levels **	11.2 (9.0,13.9)	3056	7.9 (6.6,9.6)	2401
Proportion of those eligible (aged 30 years and over) reporting that their Blood Glucose was measured by doctor/nurse/other medical personnel in the previous year**	86.2 (84.9, 87.4)	3393	78.3 (75.9,80.6)	2975
Of those whose Blood Glucose was measured in the previous year, proportion of individuals (aged over 30) reporting High Blood Glucose levels	8.8 (6.9,11.0)	2985	8.6 (7.2,10.4)	2346

*Note*: all data above are self-reported, ** indicates *p* < 0.001 for differences by sex

In an ancillary analysis (see Additional file 3, Table 1), we also found that overall, more than half the testing was happening in the private sector, with the public primary care level accounting for less than a fifth of tests in the past year for both sexes.

We also explored medication use among those tested for BP/BG and we found significant sex differences (see Additional file 3, Table 2). Of those having BPT in the previous year, 9.5% of women and 6.5% of men were on medication (this represented 83.1% of women and 80.2% of men who self-reported HBP) while 7.7% of both men and women reporting BGT the previous year reported being on medication (87.6% of women and 87.0% of men with HBG were on medication). This suggests that there may have been a shortfall in medication use of up to 19.8% to of those who already knew they had risk factors. In some cases, lifestyle modification options may have been employed for these individuals and medication may not have been required. Consequently, our sample sizes were too small to examine differences in self-reported medication use. In addition to this, we sought to explore whether women surveyed were undergoing BPT and BGT as part of their antenatal screening and coverage levels may reflect these encounters rather than general population-level testing. We computed the proportion of women who reported BPT and BGT who also reported delivering a child any time within the past 2 years – the proportions were 2.0% for BPT and 2.1% for BGT. It was therefore unlikely that antenatal testing accounted for the bulk of this testing.

### Inequalities in coverage of blood pressure testing (BPT), blood glucose testing (BGT), and self-reported prevalence of high blood pressure (HBP) and high blood glucose (HBG)

Table 3 presents summary measures of inequality for ordered and unordered dimensions in BPT coverage. Women faced education related inequality in BPT coverage, with testing being concentrated among less educated (W_SII_: -12.23; 95% CI: − 16.63, − 7.84) and wealthier populations (W_SII_: 6.67 95% CI:3.08,10.26). This inequality was of a very small magnitude in the relative measure for education (W_RCI_: -1.62; 95% CI: − 1.65, − 1.60) suggestive of concentration of testing coverage among less educated women, and by wealth (W_RCI_:1.18; 95%CI: 1.15,1.20). Men did not report significant education related inequality as reported by the SII; there were however greater BPT among wealthier groups, with a SII magnitude more than double that of women (M_SII_:14.9: 95%CI:9.9,19.9), although the difference was not statistically significant. Education (M_RCI:_0.1; 95%CI:0.08,0.11) and wealth-related inequality (M_RCI_: 2.94; 95% CI:2.86,3.02) appeared to be significantly greater among men than women in our relative measure, again, suggestive of greater testing among more educated men and wealthier men. Concentration curves for wealth and education are presented in Additional file 5.

**Table 3:** Complex Summary measures of inequality in BPT coverage for men and women

		Women (*N* = 3393)		Men (*N* = 2975)	
Blood Pressure testing		Absolute Measure	Relative Measure	Absolute Measure	Relative Measure
O^a^	Education	-12.23 (−16.63, − 7.84)	-1.62 (− 1.65, − 1.6)^b^	0.71 (− 5.02,6.45)	0.1 (0.08,0.11) ^b^
	Wealth	6.67 (3.08,10.26)	1.18 (1.15,1.2)^b^	14.9 (9.9,19.9)	2.94 (2.86,3.02)^b^
U^a^	Caste and Tribal Group	0.73 (0.3,1.6)	0.81 (0.33,1.77)	1.41 (0.69,2.9)	1.75 (0.85,3.59)
	Religion	1.24 (0.62,1.97)	1.38 (0.68,2.19)	0.33 (0.17,1.54)	0.41 (0.2,1.91)

^a^The absolute measure reported for the O or ordered dimensions (Education and Wealth) is the Slope Index of Inequality and for the U or unordered dimensions (Caste and Religion) is the Mean Difference from Mean, weighted. The relative measure reported for the O or ordered dimensions (Education and Wealth) is the Relative Concentration Index and for the U, or unordered dimensions (Caste and Religion) is the Weighted Index of Disparity. Values in parentheses are 95% confidence intervals

^b^indicates significant difference by sex

Absolute and relative caste and religion related inequalities in BPT coverage were seen among both women (Caste: W_MDM_: 0.73; 95% CI:0.3,1.6; Religion: W_MDM_: 1.24; 95% CI:0.62,1.97) and men (Caste: M_MDM_: 1.41; 95% CI:0.69,2.90; Religion: M_MDM_: 0.33; 95% CI:0.17,1.54). Overall, we found that the magnitude of inequalities by caste and religion in BPT – using absolute and relative measures - were not significantly different among women as compared to men.

Table 4 presents summary measures of inequality in self-reported HBP prevalence. Women faced significantly greater education related inequality in HBP than faced by men with almost double the inequality (as measured by SII: W_SII_: -16.79; 95% CI: − 21.22,-12.36 vs M_SII_: -7.72; 95%CI: − 12.08,-3.35). Inequality in self-reported prevalence of HBP favoured more educated populations. Interestingly, our relative measure reflected a reverse pattern: HBP self-reported prevalence was concentrated among less educated populations (W_RCI_: -20.61;95%CI: − 24.32,-16.9; M_RCI_: -12.01; 95%CI:-14.17,-9.85). Magnitude of wealth related inequality in HBP using our relative measure was significantly greater among women (W_RCI_: 9.32; 95%CI: 7.87,10.77) than among men (M_RCI_: 0.37; 95%CI:0.24,0.5), although for both, HBP was concentrated in wealthier populations (significantly more so for women). In contrast, absolute wealth related inequality in self-reported HBP prevalence was concentrated among poorer women (W_SII_: 6.5 95%CI:2.51,10.5). As depicted by the absolute summary measure weighted mean difference from mean, there was inequality in HBP self-reported prevalence by caste and tribal status, as well as different religious subgroups of women (Caste: W_MDM_: 0.8; 95% CI: 0.33,2.44; Religion: W_MDM_:3.22; 95%CI: 1.45,4.92) and men (Caste: M_MDM_: 2.06; 95% CI: 0.93,3.27; Religion: M_MDM_: 2.22; 95%CI: 1.19,3.74). Using the relative measure – weighted index of disparity, the magnitude of caste related inequality among men (M_IDIS_W_: 25.83;95%CI: 11.61,41.03) was higher when compared to that faced by women (W_IDIS_W_: 7.20; 95% CI: 3.02,22.04), though not statistically significant. Overall, we found that the magnitude of inequalities by caste and religion in self-reported HBP prevalence – using absolute and relative measures – were of significant magnitude but did not appear to vary significantly by sex.

**Table 4:** Complex Summary measures of inequality in self-reported HBP prevalence for men and women

		Women (*N* = 3056)		Men (*N* = 2401)	
Self-reported High Blood Pressure Prevalence		Absolute Measure	Relative Measure	Absolute Measure	Relative Measure
O^a^	Education	-16.79 (−21.22,-12.36) ^b^	-20.61 (−24.32, − 16.91) ^b^	−7.72 (−12.08, −3.35) ^b^	− 12.01 (−14.17, −9.85) ^b^
	Wealth	6.5 (2.51,10.5)	9.32 (7.87,10.77) ^b^	0.27 (−3.55,4.1)	0.37 (0.24,0.5) ^b^
U^a^	Caste and Tribal Status	0.80 (0.33,2.44)	7.2 (3.02,22.04)	2.06 (0.93,3.27)	25.83 (11.61,41.03)
	Religion	3.22 (1.45,4.92)	28.83 (13.03, 44.08)	2.22 (1.19,3.74)	27.96 (14.98,46.97)

^a^ The absolute measure reported for the O, or ordered dimensions (Education and Wealth) is the Slope Index of Inequality and for the U or unordered dimensions (Caste and Religion) is the Mean Difference from Mean, weighted. The relative measure reported for the O or ordered dimensions (Education and Wealth) is the Relative Concentration Index and for the U, or unordered dimensions (Caste and Religion) is the Weighted Index of Disparity. Values in parentheses are 95% confidence interval upper and lower bounds

^b ^indicates significant difference by sex

Table 5 presents summary measures of inequality for ordered and unordered dimensions in BGT coverage. Overall, we found that the magnitude of inequalities by education and wealth in BGT – using absolute and relative measures – were significantly different among women as compared to men. Women reported significantly greater education related absolute and relative inequality in BGT coverage as compared to men, with testing being concentrated among those with less education (W_SII_: -12.74; 95% CI: − 17.55, − 7.93 and W_RCI_: -1.86;95%CI: − 1.89, − 1.84 as compared to M_SII_:-0.42; 95% CI: − 6.44, 5.6 and M_RCI_: −.08; 95%CI:- 0.09,-0.07). Both sexes reported wealth related inequality in BGT with more testing in wealthier groups, although men had an SII magnitude more than double that of women (M_SII_:17.34; 95%CI:12.15,22.54; W_SII_: 7.49: 95% CI:3.36,11.63). This trend was observed in our relative measure ((W_RCI_: 1.39;95%CI: 1.36,1.41, M_RCI_: 3.55 95% CI;3.45,3.66), again, suggestive of greater testing reported among wealthier populations. Caste and religion related inequality were apparent among women (Caste: W_MDM_: 0.93; 95% CI:0.45,1.95; Religion: W_MDM_: 1.45; 95% CI:0.45,2.71) and men (Caste: M_MDM_: 1.19; 95% CI:0.92,2.43; Religion: M_MDM_: 0.75; 95% CI:0.29,2.59) although the magnitudes were not high (as was the case with BPT). For these dimensions of inequality, both absolute and relative measures were suggestive of no sex related inequality.

**Table 5:** Complex Summary measures of inequality in BG testing for men and women

		Women (*N* = 3393)		Men (*N* = 2975)	
Self-reported Blood Glucose testing		Absolute Measure	Relative Measure	Absolute Measure	Relative Measure
O^a^	Education	-12.74 (−17.55, −7.93)^b^	-1.86 (− 1.89, − 1.84) ^b^	-0.42 (− 6.44,5.6)^b^	− 0.08 (− 0.09,-0.07)^b^
	Wealth	7.49 (3.36,11.63) ^b^	1.39 (1.36,1.41) ^b^	17.34 (12.15,22.53)^b^	3.55 (3.45,3.66) ^b^
U^a^	Caste	0.93 (0.45,1.95)	1.08 (0.53,2.26)	1.19 (0.82,2.43)	1.51 (1.04,3.1)
	Religion	1.45 (0.45,2.71)	1.68 (0.53,3.14)	0.75 (0.29,2.59)	0.96 (0.37,3.31)

^a^ The absolute measure reported for the O, or ordered dimensions (Education and Wealth) is the Slope Index of Inequality and for the U or unordered dimensions (Caste and Religion) is the Mean Difference from Mean, weighted. The relative measure reported for the O or ordered dimensions (Education and Wealth) is the Relative Concentration Index and for the U, or unordered dimensions (Caste and Religion) is the Weighted Index of Disparity. Values in parentheses are 95% confidence interval upper and lower bounds

^b^ indicates significant difference by sex

Table 6 presents summary measures of inequality for ordered and unordered dimensions in self-reported HBG prevalence. Women faced significant absolute education related inequality in self-reported HBG prevalence, with prevalence concentrated among more educated populations (W_SII_: -8.02; 95%CI: − 11.96,-4.07) but our relative measure reflected the concentration of HBG prevalence among less educated populations (W_RCI_: -12.41; 95%CI: − 14.72, − 10.09). Again, this pattern of inequality was reversed among men; i.e. Men had greater self-reported HBG prevalence among less educated groups using absolute measure (M_SII_: 7.23;95%CI:2.18,12.28 and among more educated groups using our relative measure (M_RCI_: 9.73; 95%CI:8.25,11.21)). Wealth-related inequality in self-reported HBG prevalence among men was concentrated among the worse off populations (M_SII:_ 4.77; 95%CI:0.7,8.85 using our absolute measure of inequality. Interestingly, wealth-related inequality in self-reported HBG prevalence was concentrated among wealthier populations using relative measures and was significantly higher among men (M_RCI_: 8.37; 95%CI:6.79,9.94) than women (W_RCI_: 0.7 95%CI: 0.54,0.85, where the magnitude was very low). Small magnitudes of caste and religion related inequality using both absolute and relative measures (except for our relative measure for Religion, where magnitude of inequality was high but sex-related inequality was absent) were found among women (Caste: W_MDM_: 0.46; 95% CI:0. 0.36,1.98; Religion: W_MDM_: 2.19; 95%CI:0.9,4.1) and men (Caste: M_MDM_: 0.38; 95% CI:0.28,1.52; Religion: M_MDM_: 0.28 95%CI: 0.14,1.71). These differences did not appear to be statistically significant by sex.

**Table 6:** Complex Summary measures of inequality in self-reported HBG prevalence for men and women

		Women (*N* = 2985)		Men (*N* = 2346)	
Self-reported High Blood Glucose prevalence		Absolute Measure	Relative Measure	Absolute Measure	Relative Measure
O^a^	Education	-8.02 (−11.96, −4.07) ^b^	-12.41 (− 14.72, −10.09) ^b^	7.23 (2.18,12.28)^b^	9.73 (8.25,11.21)^b^
	Wealth	0.45 (−3.14,4.04)	0.7 (0.54,0.85)^b^	4.77 (0.7,8.85)	8.37 (6.79,9.94)^b^
U^a^	Caste	0.46 (0.36,1.98)	5.22 (4.07,22.44)	0.38 (0.28,1.52)	4.34 (3.22,17.51)
	Religion	2.19 (0.9,4.1)	25 (10.31,46.79)	0.28 (0.14,1.71)	3.23 (1.63,19.77)

^a^ The absolute measure reported for the O, or ordered dimensions (Education and Wealth) is the Slope Index of Inequality and for the U or unordered dimensions (Caste and Religion) is the Mean Difference from Mean, weighted. The relative measure reported for the O or ordered dimensions (Education and Wealth) is the Relative Concentration Index and for the U, or unordered dimensions (Caste and Religion) is the Weighted Index of Disparity. Values in parentheses are 95% confidence interval upper and lower bounds

^b^ indicates significant difference by sex

## Discussion

Our study assessed sex inequalities in self-reported BP and BG testing and self-reported prevalence of two NCD risk factors i.e. for HBP and HBG in Kerala. We observed sex differences in education and wealth related inequalities in BP and BG testing. We did not observe sex differences in caste and tribal status or religion-related inequalities in BP and BG testing or HBG; however magnitudes of inequality were quite high (especially for relative religion-related inequality in HBP and HBG among women).

Interestingly, there were varying patterns of education related inequality for absolute and relative summary measures for BP as compared to BG, and for testing as compared to self-reported (provider-diagnosed) prevalence of HBP and HBG. This may have had to do with the distribution of education, where there were small samples in primary education leading to a negative skew in distribution across the population. For caste and religion, given smaller sample sizes for certain groups, the confidence intervals for sex related inequalities tended to be wide as compared to within subgroup inequalities in both sexes separately. This is part of why fewer sex-related significant differences may have been found. This would have to be explored by way of analysis with larger subgroup sample sizes.

Our study found that more than three fourths of the population aged above 30 reported getting tested for BP and BG in the previous year. A study in Kasaragod, Kerala (one of the sampled districts in our study), with a sample size of 375 respondents aged 30 years and over found that over 62% had undergone NCD screening [16]. Given such relatively high rates of screening, high testing rates in our study may be related to the prior exposure to the health system. This exposure is gendered: a 1993 study in the US examining factors associated with health screening among women of reproductive age group found this to be the case [39]. Following from this, higher BP and BG testing among women could also be because women usually get screened during their pregnancy to screen for pre-eclampsia; this has lifetime implications for management of chronic disease [40]. There is clearly a closer need to understand why this sex difference was found in our study, how it may relate to population-based screening, and whether it reflects a broader pattern or not.

Our study found that provider-diagnosed prevalence of HBP, not HBG, was higher among women than men. These sex differences in self-reported prevalence of HBP and HBG have also been reported in other studies conducted in Kerala and India. For instance, a cross sectional survey conducted in 2016 among 1154 adults (above 30) found that the prevalence of hypertension was higher among women compared to men [41]. Furthermore, results from NSSO data (71st round, survey data) examining self-reported prevalence of NCDs in India and Kerala also found higher NCDs risk among women than men [3]. Although, women in developing country settings tend to report their symptoms more than men, leading to a higher prevalence rate of self-reported diseases as compared to men [3], we found not sex difference in self-reported HBG prevalence.

Interestingly, a newly released NFHS-5 fact sheet for Kerala, 2019 reported that the proportion of women with mildly and moderately or severely HBP were 15.5 and 6.6% respectively; these values were 19.2 and 6.7% among men. In our study, the self-reported prevalence of HBP among women and men was 11.2 and 7.9% respectively. This seems to suggest undetected HBP, although given the current unavailability of raw NFHS 5 data, we are not able to ascertain if our values were significantly different. Regarding BG, NFHS 5 reported that the proportion of women with “high” (141–160 mg/dl) and “very high” (> 160 mg/dl) BG levels was 8.3 and 13.1% respectively; the values for men were 9.8 and 13.8% respectively. In our survey, HBG was reported by 8.8% of women and 8.6% of men, but it is unclear if self-reported data is indicative of clinically measured “high “or “very high” levels. However, the lack of sex difference was also seen in our study.

Our study suggests that education and wealth related inequalities exist in testing for BP and BG as well as self-reported prevalence of HBP and HBG among both men and women. Sex differences in socio-economic patterns of NCD testing and prevalence have been found globally. A European study examining educational inequalities in the use of BP and cholesterol screening in nine countries found a positive gradient (i.e. greater screening among higher educated groups) with Hungary being the only country where the least educated group was more likely to be screened [25]. The study attributed the higher rate to the effect of general health: screening rates were higher among those with poor health and poor health is concentrated among lower socioeconomic groups [25]. A Japanese study examining educational inequalities in NCD incidence using data from a longitudinal survey of middle aged respondents found lower education level was positively associated with diabetes incidence in both genders, but with hypertension only among women [42].

We found pro-rich inequalities across men and women, in which wealthier groups had greater access to testing with significant sex differences depicting higher inequality among men than women. Varying patterns for wealth and education, suggests they are not collinear – i.e. there may be lower income males with higher education who may be missing out on testing. More granular data powered for intersectional analysis of wealth by education could help unravel the cause of such varying patterns. Wealth related inequality in HBG showed significant sex differences with greater inequality among men and HBG concentration among the wealthier groups. Similar to our findings, a study conducted in Kerala 2012 reported that the proportion of self-reported diabetes was highest (at 11.1%) in the group with the highest socioeconomic status, when compared with 3.1% in lower socioeconomic positioned groups. Similar proportions were observed in both sexes [20].

In our study, absolute and relative measures reflected distinct patterns of inequality where on one hand, using our absolute measure HBP prevalence was concentrated among more educated and lower income groups, on the other hand, the relative measure showed the opposite: concentration among higher-income and less-educated groups. Additionally, using the absolute measure, self-reported HBG prevalence was also concentrated among more-educated women but a reversed scenario among men, it was concentrated among less-educated populations, but again our relative measure displayed an opposite pattern. These findings reflect that the education and wealth inequalities in self- reported HBP and HBG differ using absolute and relative measures of inequality. Other similar studies have also found contrasting magnitude and directions of inequalities using absolute and relative measures [43–45] reflecting the importance of reporting both summary measures. SII is sensitive to variation in distribution of population among different socioeconomic groups [46]: Illiterate population in our sample was low which might have resulted in a biased estimate. Similar to our findings for relative measures, a national study using NSSO 71st round data found that prevalence of NCDs was reportedly higher among illiterate or low educated women as compared to men with same education level [3]. Further, a paper using the study on Global Ageing and Adult Health, 2007 of 12,198 adult respondents found that self-reported and clinically measured hypertension were concentrated among the affluent and educated in India. This paper also found that the magnitude of inequality using standardised measures was much lower when compared to the magnitude of inequality using self-reported measures indicating under-diagnosis and under-reporting among poor [22].

We found that sex-related inequalities by religion and caste in testing and prevalence of NCD risk factors (HBP and HBG) were not statistically significant. Testing coverage was highest among women belonging to ‘other backward class group’. Magnitudes of inequality in HBP and HBG by caste and tribal status, particularly using our relative measure, were high for both sexes. Studies have found that NCD mortality and the prevalence of risk factors are high among tribal populations. A study on Kani tribal groups in Kerala found out that prevalence of hypertension was higher among Kani tribes-people when compared to general population [18]. A national study examining gender difference in the prevalence of NCDs drawing NSSO 71st round data found that prevalence of NCDs was higher among women from illiterate, OBC and other castes, Christians and wealthier groups when compared to their men counterparts [3].

Our study had some major limitations. Firstly, we relied on self-reported data to estimate the prevalence of HBP and HBG rather than clinical measurement. Results from self-reported health illness in sample surveys (small or large) should be interpreted with caution, as they can – and in our case likely - underestimate prevalence [24]. Secondly, our data did not include younger respondents (below 30 years). However, this would have a limited impact on the findings, as most of the conditions we studied occur largely in adults, 15 million of 41 million deaths are due to NCDs between the ages of 30 and 69 years [47]. Thirdly, while conducting the survey, sometimes participants reported on behalf of other members who were not present in the house during survey. So, this may have under/overestimated the results. Lastly, we had 13 individuals in our sample overall who self-identified as transpersons. We were not able to explore/analyse inequalities comparing this group using three groups or other forms of gender analysis or disaggregation given the limitations of the frequentist approach used in our analysis. This should certainly be explored in the future.

Our study suggests complex patterns of self-reported testing for BP and BG in Kerala. Wealthier populations seem to have had greater access to testing overall. The relationship of educational status to testing appears to be vexed, however – this could be because very small proportions of the population are uneducated – but in relative terms, less educated men and women do seem to be left out from testing. Targeted testing for populations with low literacy may be considered. Moreover, policies may consider education around NCD risk factors across religious groups because it seems that Muslim and Hindu populations have greater testing (as seen in National Family Health Survey data as well). There is some indication that working with religious and community leaders can yield greater community-level buy in. Further research and policy should examine NCD risk factor service coverage and prevalence across genders (beyond cis categories). Regular clinical measurement of BP and BG alongside information on testing in the system will also be necessary to determine what is the unmet need for testing as well as screening and how ongoing programs are faring at filling gaps in outreach. There are ongoing studies in Kerala of this nature, it is possible that if data from these studies is pooled, it can be made readily available to link up to decision-making with a focus on inclusion.

## Conclusion

We found sex related inequalities by wealth and education in both testing indicators and self-reported prevalence indicators for blood pressure and blood glucose. High income groups reported higher levels of testing and provider diagnosed prevalence of high blood pressure and blood glucose. Magnitude of wealth-related inequalities were greater among men than women overall except for prevalence of high blood pressure, where relative inequalities were greater among women. In relative terms, educational attainment and wealth seem to be associated with greater testing while self-reported prevalence appears to vary by religious groups. These patterns require further exploration to understand contexts and pathways to ensure program design leaves no one behind.

## Supplementary Information

**Additional file 1.** Descriptive analysis and simple measures of inequality for men and women in each of the four selected indicators.

**Additional file 2.** Definitions and Interpretations of the summary measures used to measure inequality in each of the four selected indicators.

**Additional file 3.** Additional information on selected NCD risk indicators.

**Additional file 4.** Additional information on the study design, sampling methodology and data collection.

**Additional file 5.** Concentration Curves for Blood Pressure and Glucose testing and self-reported prevalence for High Blood Pressure and Glucose by education and wealth.

## Data Availability

All datasets used for supporting the conclusions of this paper are available from the corresponding author on request.

## Abbreviations

NCDs: Non-Communicable Diseases
BP: Blood Pressure
BG: Blood Glucose
RCI: Relative, Concentration Index
BPT: Blood Pressure Screening
BGT: Blood Glucose Screening HBP: High Blood Pressure
HBG: High Blood Glucose
CVD: Cardiovascular diseases
NPCDCS: National Programme for Prevention and Control of Cancer, Diabetes, CVD and Stroke
SDGs: Sustainable Development Goals
FHC: Family Health Centre
PHC: Primary Health Centre
PSU: Primary Sampling Unit
NSSO: National Sample Survey Office
SII: Slope Index of Inequality
MDM: Weighted Mean difference from Mean
IDIS_W: Weighted Index of Disparity
NFHS: National Family Health Survey
Lb: Lower Bound
Ub: Upper Bound

## Footnotes

1 Survey weights were used to cater for different selection probabilities in different blocks of the survey and to adjust for household non-response and individual non-response. Survey weights were calculated based on sampling probabilities separately for each sampling stage. The final sampling weights were normalized in order to give a total number of weighted cases that equals the total number of unweighted cases. Normalization was done by multiplying the sampling weight by the estimated total sampling fraction obtained from the survey for household weights and individual weights.

2 For caste and religion groups, we carried out a separate analysis by grouping ‘general’ caste category with prefer not to say/don’t know as well as by grouping ‘other religious group’ with prefer not to say/don’t know. In all analyses, results were similar to those presented here.
