# Supplementary material for: Intersecting sex-related inequalities in self-reported testing for and prevalence of Non-Communicable Disease (NCD) risk factors in Kerala
Source: BMC Public Health. 2022 Mar 19;22:544. doi: 10.1186/s12889-022-12956-w (PMC8933933; doi:10.1186/s12889-022-12956-w)
Supplement: Supplementary file 1 — Additional file 1. Descriptive analysis and simple measures of inequality for men and women in each of the four selected indicators. [file 12889_2022_12956_MOESM1_ESM.docx]

**Descriptive analysis and simple measures of inequality for men and women in each of the four selected indicators**

### **Sex differentials in Blood Pressure testing (BPT) coverage and Self-reported prevalence of High Blood Pressure (HBP)**

Table S1 shows BPT coverage had a statistically significant association with education, wealth, and religion groups among women (p<0.001). However, among men, BPT had a statistically significant association with caste groups and wealth. Additionally, there were significant sex differences across subgroups of education, wealth, religion, and caste/tribal status in BPT coverage (see Table S1). Within subgroups, BPT coverage was significantly higher among women than men across all education categories except primary. BPT coverage followed a wealth gradient among both men and women, except in the richest quintile. BPT coverage was significantly higher among Hindu (BPT_W_:90.6% vs BPT_M_:80.5%), Muslim (BPT_W_:92.4% vs BPT_M_:81.3%) and Christian women (BPT_W_:86.5% vs BPT_M_:81.2%) as compared to their men counterparts (with p<.001). BPT coverage was significantly higher among tribal (BPT_W_:82.5% vs BPT_M_:59.5%), OBC (BPT_W_:90.9% vs BPT_M_:80.3%) and general caste (BPT_W_: 90.3% vs BPT_M_:83.1%) women as compared to men. Absolute sex-related inequality in BPT coverage was highest in Scheduled Tribes (23 percentage points) followed by Illiterate persons (22 percentage points) favouring women in both subgroups. Relative sex related simple inequality between subgroups was also highest in these subgroups. Sex-related inequality in BPT coverage by simple measures was lowest among those educated till primary and the richest with a difference of less than five percentage points and a ratio of 1 depicting no difference in BPT coverage among men and women in these subgroups.

Table S1: BPT coverage for men and women across dimensions of inequality

| **Dimensions** | BPT coverage | | | |
| --- | --- | --- | --- | --- |
|  | Women | Men | Between Sex Difference | Between Sex Ratio |
|  | mean (95%CI) | (mean (95%CI) |  |  |
| **Level of Education** | **##** |  |  |  |
| Illiterate ** | 92.3 (83.9,96.5) | 70.3 (52.9,83.3) | 22.0 | 1.3 |
| Primary | 90.3 (74.0,96.8) | 88.7 (72.3,95.9) | 1.6 | 1.0 |
| Secondary** | 94.2 (92.7,95.4) | 81.6 (77.4,85.1) | 12.6 | 1.2 |
| Higher Secondary and above** | 87.4 (85.5,89.2) | 80.8 (78.0,83.3) | 6.7 | 1.1 |
| **Wealth** |  | **##** |  |  |
| Poorest (Quintile 1)** | 87.0 (83.5,90.5) | 75 (67.7,82.3) | 11.9 | 1.2 |
| Poor (Quintile 2)** | 88.8 (85.2,92.4) | 78.1 (74.2,81.9) | 10.7 | 1.1 |
| Middle (Quintile 3)** | 91.4 (88.6,94.3) | 79.8 (74.7,84.9) | 11.7 | 1.1 |
| Richer (Quintile 4)** | 91.2 (88.5,94) | 82.5 (77.3,87.7) | 8.7 | 1.1 |
| Richest (Quintile 5)* | 92.5 (89.7,95.3) | 88.0 (84.6,91.3) | 4.6 | 1.1 |
| **Religion** | ## |  |  |  |
| Hindu** | 90.6 (89.3,91.9) | 80.5 (78.2,82.8) | 10.1 | 1.1 |
| Muslim ** | 92.4 (90.7,94.2) | 81.3 (75.8,86.8) | 11.1 | 1.1 |
| Christian** | 86.5 (82.3,89.8) | 81.2 (76.4,85.1) | 5.3 | 1.1 |
| **Caste and tribal status** | # | ## |  |  |
| Scheduled Caste (SC) | 87.9 (82.1,92.1) | 81.5 (73.8,87.3) | 6.4 | 1.1 |
| Tribal Status (ST)** | 82.5 (74.4,88.5) | 59.5 (44.4,73.0) | 23.0 | 1.4 |
| Other Backward Class (OBC)** | 90.9 (89.6,92.1) | 80.3 (77.8,82.5) | 10.7 | 1.1 |
| General** | 90.3 (88.5,91.9) | 83.1 (79.8,86) | 7.2 | 1.1 |

Note: ** and * indicates p<0.001and p<0.05 for differences by sex, ## indicates p<.05 for within group differences separately for men and women, values rounded to one digit.

Table S2 shows that there was a statistically significant association between self-reported prevalence of HBP with education and religion among both sex and by wealth among women. Within subgroups, findings show that self-reported HBP was significantly greater among women as compared to men concentrated among the most educated (HBP_W_:7.3:95% CI:5.2,10.0) ; HBP_M_: 6.6:95% CI:5.1,8.5), fifth wealth quintile (HBP_W_:16.1;95% CI :11.5,20.7; HBP_M_:8.8;95% CI :5.4,12.2) , Muslim (HBP_W_:17.2:95% CI:11.5,22.9) ; HBP_M_:11.8:95% CI:7,16.1) and among general caste category (HBP_W_:11.5;95% CI:8.3,15.8; HBP_M_:5.0;95% CI: 2.8,8.7). Sex-related Inequality (last two columns in table 4) reflected highest absolute difference in self-reported HBP prevalence of 7.6 percentage points within richest quintile followed by 6.5 percentage points in general category and lowest of 0.04 percentage points among schedule caste group and 0.7 the most educated group (higher among the women in these groups). However, relative sex-related inequality reflected a different scenario: highest ratio of 2.3 in general caste group followed by 1.8 in the richest quintile and lowest (1.1) in the most educated group.

Table S2: Prevalence of self-reported HBP for men and women across dimensions of inequality

| **Dimensions** | Prevalence of HBP (mean,95%CI) | | | |
| --- | --- | --- | --- | --- |
|  | Women (N=3036) | Men (N=2388) | Between Sex Difference | Between Sex Ratio |
|  | (mean,95%CI) | (mean,95%CI) |  |  |
| **Level of Education** | **##** | **#** |  |  |
| Illiterate | 20.4 (14.1,28.5) | 16.6 (7.2,33.9) | 3.8 | 1.2 |
| Primary | 20.5 (10.3,36.7) | 20.2 (4.0,60.9) | 0.3 | 1.0 |
| Secondary | 14.4 (10.5,19.3) | 9.3 (6.7,12.6) | 5.1 | 1.6 |
| Higher Secondary and above** | 7.3 (5.2,10.0) | 6.6 (5.1,8.5) | 0.7 | 1.1 |
| **Wealth** | **#** |  |  |  |
| Poorest (Quintile 1) | 9 (3.8,14.1) | 8.1 (3.5,12.7) | 0.9 | 1.1 |
| Poor (Quintile 2) | 10.1 (6.9,13.4) | 7 (4.1,10) | 3.1 | 1.4 |
| Middle (Quintile 3) | 11.4 (7.3,15.6) | 9.8 (6.5,13.1) | 1.6 | 1.2 |
| Richer (Quintile 4) | 8.3 (5.4,11.2) | 5.4 (2.4,8.3) | 2.9 | 1.5 |
| Richest (Quintile 5)** | 16.1 (11.5,20.7) | 8.8 (5.4,12.2) | 7.3 | 1.8 |
| **Religion** | ## | # |  |  |
| Hindu | 8.7 (6.7,10.7) | 6.3 (4.7,7.8) | 2.4 | 1.4 |
| Muslim * | 17.2 (11.5,22.9) | 11.8 (7,16.7) | 5.3 | 1.5 |
| Christian | 14.1 (10.0,19.6) | 10.5 (7.3,14.9) | 3.6 | 1.4 |
| **Caste and tribal status** |  | # |  |  |
| Scheduled Caste (SC) | 6.3 (2.5,15.1) | 6.3 (3.3,12) | 0.0 | 1.0 |
| Tribal Status (ST) | 8.4 (2.1,28) | 2.4 (0.4,13.2) | 6.0 | 3.5 |
| Other Backward Class (OBC) | 11.5 (9.4,14) | 9.6 (7.9,11.6) | 1.9 | 1.2 |
| General** | 11.5 (8.3,15.8) | 5 (2.8,8.7) | 6.5 | 2.3 |

Note: ** indicates p<0.001 and * indicates p<0.05 for differences by sex, ## indicates p<.001 and # indicates p<.05 for within group differences separately for men and women. Values rounded to one digit

### **Sex differentials in Self-reported testing of Blood Glucose (BGT) and Self-reported prevalence of High Blood Glucose levels (HBG)**

Table S3 shows that BGT coverage had statistically significant associations with education groups among women. In addition, BGT coverage had significant association with wealth and caste groups among men. Further, the findings show sex differentials in BGT coverage disaggregated by education, wealth, religion and caste/tribal status within subgroups. The overall trend was similar to the trends seen for BPT. Within subgroups, BGT was significantly higher among women than men across all education categories except primary. Women were tested in significantly greater proportions than men across three bottom wealth quintiles except the rich and richest quintile. BGT coverage was significantly higher among Hindu (BGT_W_:87.1% vs BGT_M_:78.1%), Muslim (BGT_W_:86.9% vs BGT_M_:80.4%) and Christian (BGT_W_:81.7% vs BGT_M_:76.7%) women than their men counterparts. BGT coverage was significantly higher among tribal (BGT_W_:79.2% vs BGT_M_:57.5%), OBC (BGT_W_:86.1% vs BGT_M_:78.3%) and general (BGT_W_:87.9% vs BGT_M_:80.5%) women than men except ‘Schedule Caste’ category. Sex-related inequality in BGT coverage according to absolute difference was about 22 percentage points among illiterate and ST population (favouring women) and lowest among primary education and top two wealth quintiles (Difference:5 and ratio 1.1).

Table S3: BGT coverage for men and women across four dimensions of inequality

|  | BGT coverage (mean, 95%CI) | |  |  |
| --- | --- | --- | --- | --- |
|  | Women | Men | Between Sex Difference | Between Sex Ratio |
| **Education** | **##** |  |  |  |
| Illiterate** | 90.1 (82.7,94.6) | 67.9 (53.2,79.7) | 22.3 | 1.3 |
| Primary | 88.9 (73.9,95.8) | 92 (76.5,97.6) | 3.0 | 1.0 |
| Secondary** | 89.7 (86.8,92) | 79.3 (75.2,82.9) | 10.4 | 1.1 |
| Higher Secondary & above** | 83.3 (81.8,84.6) | 78.1 (75.4,80.7) | 5.1 | 1.1 |
| **Wealth** |  | **##** |  |  |
| Poorest (Quintile 1)** | 82.3 (78.9,85.8) | 70.8 (63.8,77.8) | 11.5 | 1.2 |
| Poor (Quintile 2)** | 84.5 (80.4,88.7) | 75.6 (70.9,80.3) | 8.9 | 1.1 |
| Middle (Quintile 3)** | 88.5 (85.5,91.6) | 77.3 (72.3,82.4) | 11.2 | 1.1 |
| Richer (Quintile 4) | 84.7 (81,88.4) | 81 (75.7,86.3) | 3.7 | 1.0 |
| Richest (Quintile 5) | 89.7 (86.9,92.5) | 86.1 (82.6,89.5) | 3.7 | 1.0 |
| **Religion** | **#** |  |  |  |
| Hindu** | 87.1 (85.5,88.7) | 78.1 (75.7,80.6) | 9.0 | 1.1 |
| Muslim* | 86.9 (83.1,90.7) | 80.4 (74.7,86.1) | 6.5 | 1.1 |
| Christian* | 81.6 (76.0,86.2) | 77.0 (71.3,81.7) | 4.7 | 1.1 |
| **Caste and tribal status** |  | **##** |  |  |
| Schedule Caste | 83.4 (75.7,89) | 75.7 (66.7,82.9) | 7.7 | 1.1 |
| Tribal Status (ST)** | 79.2 (73.3,84.2) | 57.3 (44,69.6) | 22.0 | 1.4 |
| OBC** | 86.1 (84.3,87.7) | 78.3 (75.5,80.9) | 7.8 | 1.1 |
| General ** | 87.7 (85.4,89.7) | 80.6 (77.4,83.5) | 7.1 | 1.1 |

Note: ** indicates p<0.001 and * indicates p<0.05 for differences by sex, ## indicates p<.001 and # indicates p<.05 for within group differences separately for men and women. Values rounded to one digit

Table S4 shows that self-reported HBG prevalence had statistically significant associations with education group among both men and women. In addition, self-reported HBG prevalence had statistically significant association with wealth groups among men and religion groups among women. Within education subgroups, HBG was significantly higher among women than men in the Illiterate group (HBG_W_: 11.6% vs HBG_M_:0.3%), primary education group (HBG_W_: 10.5% vs HBG_M_:1.9%) and the reverse in the ‘higher secondary and above’ education category (HBG_W_:6.7% vs HBG_M_:9.8%). Sex-related inequality among illiterate populations demonstrated by ratio and difference reflected a difference of 11.3 percentage points but a very high ratio of 37.9. Sex-related inequality among the most educated populations reflected a difference of 3.1 percentage points (higher among men) but a ratio of only 0.7.

Table S4: Prevalence of self-reported HBG for men and women across four dimensions of inequality

|  | Prevalence of HBG (mean, 95%CI) | | | |
| --- | --- | --- | --- | --- |
|  | Women | Men | Between Sex Difference | Between Sex Ratio |
| **Education** | **##** | **##** |  |  |
| Illiterate* | 11.6 (6,21.2) | 0.3 (0,2.7) | 11.3 | 37.9 |
| Primary** | 10.5 (4.6,22.1) | 1.9 (0.3,12.4) | 8.5 | 5.5 |
| Secondary | 10.9 (8.1,14.6) | 7.3 (5.3,10.1) | 3.6 | 1.5 |
| Higher Secondary & above** | 6.7 (4.9,9.2) | 9.8 (8.2,11.8) | 3.1 | 0.7 |
| **Wealth** |  | **#** |  |  |
| Poorest (Quintile 1) | 8.8 (4.5,13.1) | 9.6 (5.2,13.9) | 0.1 | 0.9 |
| Poor (Quintile 2) | 8.2 (5.5,11) | 5.5 (3.2,7.9) | 2.7 | 1.5 |
| Middle (Quintile 3) | 9.3 (6,12.5) | 8.4 (5.2,11.6) | 0.9 | 1.1 |
| Richer (Quintile 4) | 8.3 (5,11.5) | 6.8 (3.3,10.4) | 1.4 | 1.2 |
| Richest (Quintile 5) | 9.1 (6,12.2) | 12.3 (7,17.6) | 3.2 | 0.7 |
| **Religion** | **#** |  |  |  |
| Hindu | 7.1 (5.1,9.1) | 8.5 (6.9,10.1) | 1.4 | 0.8 |
| Muslim | 12.6 (7.7,17.5) | 8.4 (4.5,12.2) | 4.2 | 1.5 |
| Christian | 11.1 (6.4,18.6) | 9.5 (6.7,13.3) | 1.7 | 1.2 |
| **Caste and tribal status** |  |  |  |  |
| Schedule Caste | 8.7 (4.2,17.3) | 8.5 (4.2,16.4) | 0.2 | 1.0 |
| Tribal Status (ST) | 1.2 (0.1,10.1) | 3.4 (0.5,20.3) | 2.2 | 0.35 |
| OBC | 9.2 (6.9,12.1) | 8.5 (6.6,10.8) | 0.7 | 1.1 |
| General | 8.6 (6.4,11.5) | 9.3 (6.6,13) | 0.8 | 0.9 |

Note: ** indicates p<0.001 and * indicates p<0.05 for differences by sex, ## indicates p<.001 and # indicates p<.05 for within group differences separately for men and women. Values rounded to one digit
