# Supplementary material for: Intersecting sex-related inequalities in self-reported testing for and prevalence of Non-Communicable Disease (NCD) risk factors in Kerala
Source: BMC Public Health. 2022 Mar 19;22:544. doi: 10.1186/s12889-022-12956-w (PMC8933933; doi:10.1186/s12889-022-12956-w)
Supplement: Supplementary file 2 — Additional file 2. Definitions and Interpretations of the summary measures used to measure inequality in each of the four selected indicators. [file 12889_2022_12956_MOESM2_ESM.docx]

# **Definitions and Interpretations of the selected summary measures**

Slope Index of Inequality is an absolute measure of inequality, is regression based and represents the slope of regression obtained by regressing health variable of interest against weighted midpoint of cumulative population of each subgroup after ranking them from lowest to highest range using a generalized linear model with logit link. For favourable indicators like Blood Pressure Testing (BPT) and Blood Glucose Testing (BGS), positive values of SII suggest greater prevalence among the most advantaged. For unfavourable indicators like High Blood Pressure (HBP) and High Blood Glucose (HBG), positive values of SII suggest greater prevalence among the most disadvantaged while the negative values reflect the opposite: higher prevalence among advantaged. It takes the value zero in the absence of inequality [1].

Relative measures namely Relative Concentration Index (RCI) was computed for ordered dimensions: wealth and education. RCI is twice the area between concentration curve and the 45-degree line of inequality. RCI is zero in the absence of inequality, negative when concentration of health variable (like BPT/ prevalence of HBP) is concentrated among lower ranked populations (like poor, uneducated etc.) and positive when concentration of health variable is among higher ranked populations (like rich, most educated etc.). It ranges between -100 to 100 as the fraction is multiplied by 100.

Weighted Mean Difference of Mean is a weighted, complex, and an absolute measure of inequality. It is obtained by calculating sum of absolute differences between subgroups and setting average and dividing by the number of subgroups. Larger values show a larger magnitude of inequality and its value is zero in the absence of inequality [1].

Weighted Index of Disparity is a weighted, complex, relative measure of inequality. It is calculated by first obtaining sum of absolute differences between the subgroup estimates and setting average, weighing absolute differences by population share of each subgroup𝑝 and dividing it by setting average. It only takes positive values and farther the values are from zero, larger is the magnitude of inequality. Its value is zero if there is no inequality [1].

Theil Index is a relative, complex summary measures of inequality which is measured by first calculating the product of share of each population subgroup, ratio of share of the subgroup to the setting average and taking natural logarithm of this ratio. Then, the products are added to get the estimate of Theil index which is multiplied by 1000 for easier interpretation [1]. Greater absolute values suggest greater level of inequalities. Due to the use of natural logarithm in its calculations, TI is sensitive to the differences from setting average. In our study, Caste and tribal status and Religion subgroups had differences from the setting average (also due to lower sample size in these subgroups). Therefore, we did not prefer to use this summary measure. For more explanation on these summary measures, please refer to the technical notes of Health Equity Assessment Toolkit [1].

**Results using Theil Index for all four Indicators separately for men and women.**

|  | | |
| --- | --- | --- |
|  | Women | Men |
| BP Testing | | |
| Caste and Tribal Status | 0.13(0.13,0.13) | 0.86(0.86,0.86) |
| Religion | 0.21(0.21,0.21) | 0.01(0.01,0.01) |
| High BP prevalence | | |
| Caste and Tribal Status | 9.31(9.29,9.33)** | 41.12(41.07,41.18)** |
| Religion | 45.98(45.93,46.03) | 42.1(42.04,42.15) |
| BG Testing | | |
| Caste and Tribal Status | 0.17(0.17,0.17) | 0.91(0.91,0.91) |
| Religion | 0.27(0.27,0.27) | 0.1(0.1,0.1) |
| High BG Prevalence | | |
| Caste and Tribal Status | 12.99(12.98,13.01)** | 4.18(4.16,4.19)** |
| Religion | 33.93(33.88,33.98)** | 0.93(0.93,0.94)** |

** denotes significant sex differences. Values in parentheses are 95% confidence interval upper and lower bounds

**References**

1. **Health Equity Assessment Toolkit (HEAT): Software for exploring and comparing health inequalities in countries. Built-in database edition Version 2.1 Geneva, World Health Organization**. In*.*; 2018.
