# Supplementary material for: Intersecting sex-related inequalities in self-reported testing for and prevalence of Non-Communicable Disease (NCD) risk factors in Kerala
Source: BMC Public Health. 2022 Mar 19;22:544. doi: 10.1186/s12889-022-12956-w (PMC8933933; doi:10.1186/s12889-022-12956-w)
Supplement: Supplementary file 3 — Additional file 3. Additional information on selected NCD risk indicators. [file 12889_2022_12956_MOESM3_ESM.docx]

**Additional information on four selected indicators.**

**Table S5: Composition of self-reported Blood Pressure and Glucose testing in the previous year by facility type**

| **Facility type** | | **Blood Pressure testing** | | **Blood Glucose testing** | | |
| --- | --- | --- | --- | --- | --- | --- |
|  | **Women**  **Mean (95% CI)** | | **Men**  **Mean (95% CI)** | | **Women**  **Mean (95% CI)** | **Men**  **Mean (95% CI)** |
| Primary Health Centres | 15.1 (12.8,17.6) | | 11.5 (9.5,14) | | 13 (11.2,15) | 10.5 (8.7,12.7) |
| Family Health Centres | 6.2 (4.5,8.5) | | 4.4 (3.3,5.8) | | 5.6 (3.9,7.9) | 3.2 (2.6,4) |
| Other public facilities | 24.9 (22.1,28) | | 27.8 (24.9,30.9) | | 23.9 (21,27.1) | 26.9 (24.1,29.9) |
| Private | 51.6 (48.7,54.5) | | 54.4 (50.9,57.8) | | 55.3 (52.3,58.4) | 57.1 (53.8,60.4) |
| Others | 2.2 (1.5,3.3) | | 1.9 (1.3,2.9) | | 2.2 (1.5,3.2) | 2.3 (1.6,3.1) |

**Table S6:** **Indicators for self-reported Blood Pressure and Blood Glucose testing and prevalence**

|  | | **Women** | | **Men** | |
| --- | --- | --- | --- | --- | --- |
|  | **Blood Pressure (BP) related indicators** | | | |  |
|  | | **Mean (95% CI)** | **N** | **Mean (95% CI)** | **N** |
| Proportion of individuals (aged over 30) whose BP was ever measured by doctor/nurse/other medical personnel | | 96.7 (95.9,97.4) | 3393 | 90.3 (88.8,91.7) | 2975 |
| Of those whose BP was ever measured, proportion of individuals (aged over 30) who reported higher than normal BP levels | | 10.6 (8.5,13.1) | 3274 | 7.2 (6,8.6) | 2681 |
| Of those whose BP was ever measured, proportion of individuals (aged over 30) who reported they were on medication * | | 8.8 | 3274 | 5.8 | 2681 |
| Of those whose BP was measured previous year, proportion of individuals (aged over 30) who reported they were on medication* | | 9.5 | 3056 | 6.5 | 2401 |
| Of those who reported high BP, proportion of individuals (aged over 30) who reported they were on medication currently | | 83.1 (78.4,86.9) | 348 | 80.2 (72.4,86.3) | 194 |
|  | **Blood Glucose (BG) related indicators** | | | |  |
| Proportion of individuals (aged over 30) whose BG was ever measured by doctor/nurse/other medical personnel | | 94.5 (93.6,95.3) | 3393 | 88.2 (86.6,89.6) | 2975 |
| Of those whose BG was ever measured, proportion of individuals (aged over 30) who reported higher than normal BG levels | | 8.2 (6.5,10.3) | 3196 | 8 (6.6,9.7) | 2618 |
| Of those whose BG was measured, proportion of individuals (aged over 30) who reported they were on medication currently* | | 7.2 | 3196 | 6.9 | 2618 |
| Of those whose BG was measured previous year, proportion of individuals (aged over 30) who reported they were on medication * | | 7.7 | 2985 | 7.7 | 2346 |
| Of those who reported high BG, proportion of individuals (aged over 30) who reported they were on medication currently | | 87.6 (80.4,92.4) | 262 | 87 (78.8,92.4) | 209 |

A: Note that numbers used to calculate percentages were obtained in Stata, percentages may not match due to rounding of.

B: N denotes denominator.

* Confidence Intervals were not available.
