# Supplementary material for: Intersecting sex-related inequalities in self-reported testing for and prevalence of Non-Communicable Disease (NCD) risk factors in Kerala
Source: BMC Public Health. 2022 Mar 19;22:544. doi: 10.1186/s12889-022-12956-w (PMC8933933; doi:10.1186/s12889-022-12956-w)
Supplement: Supplementary file 4 — Additional file 4. Additional information on the study design, sampling methodology and data collection. [file 12889_2022_12956_MOESM4_ESM.docx]

**Additional information on the study design, sampling methodology and data collection**

The study used multistage random sampling design. Clusters of districts were made using principal component analysis. Principal Component Analysis is a dimension-reduction tool that helps in reducing a large set of variables to a smaller set containing most of the information from the larger set. Set of variables that were used for grouping these districts were household with electricity, improved drinking water, improved sanitation, clean fuel for cooking, insurance, Women with 10 or more years of schooling (%), high glucose in women and men, women who ever had cervix examination, women with below normal Body Mass Index (BMI), above normal BMI, children with full immunization, hypertension in men and women, and children reporting Diarrhoea in last 2 weeks. Using Composite factor scores (first), all 14 districts of Kerala were divided into four groups.

One district was selected from each of the four groups randomly and two facilities per district were randomly selected. The sampling frame was reviewed, and concurrence was received from state officials

**Sample Size Determination**

For this study, corresponding with our approved indicators ( published elsewhere [1]) relevant at the population level, we had four populations of interest: Infants under a year of age, Children under five, women with a live birth in the past year, and blood pressure screening/testing of adults over the age of 30. While there is fairly high-quality record keeping for the first of the three groups, little to no information on blood pressure screening was available from facility data at the population level. We, therefore, powered the study to draw estimations at this scale.

Thus, the primary outcomes of the study were

- Proportion of women of age greater than equal to 30 years who were tested for blood pressure last year in Kerala
- Proportion of men of age greater than equal to 30 years who were tested for blood pressure last year in Kerala

Sample Size was calculated using the formula

$$n=\frac{z^{2}\mathrm{pq}}{e^{2}}$$

Where n is the sample size, z2 is the abscissa of the normal curve that cuts off an area α at the tail, e is the desired level of precision, p is the expected true proportion that is present in the population, and q is 1-p. Using the above formula, desired level of precision=8% at 95% confidence interval, sample sizes for women and men were computed separately. Take a conservative design effect of 2, the sample sizes were doubled.

**Sample Selection**

We visited the health facility first to obtain any existing maps of the catchment area and if it was not available, we created one with the help of field staff of the health department. The wards in each panchayat area was mapped with roads landmarks and houses. The ward area was further detailed with the help of elected ward members with major roads, tree lines, canal, bridges, landmarks, and houses. After the creation of artificial ward segments, one or two segments were randomly selected. A route map with direction and road for the investigators were planned for each day with a prominent landmark being start point. For household selection, systematic sampling methodology was used. Sampling Interval (SI) was computed by dividing by the total number of households in each ward segment by the specified number of households to be drawn from each ward segment. A random number ( r ) was drawn between 1 and S**I.** The first household to be selected was the one with the number corresponding to this random number. After that the series of selected households were based on SI (example R+SI, R+2*SI, R+3*SI, etc. until we arrived at the specified number of households). If a house was unoccupied at the time of a visit, that house was revisited later that day. If the house was permanently vacant or if the household members declined to participate in the survey, then the next closest household was selected. In each house the head of the household was the primary respondent to the questions. Investigators had a daily household visitation log sheet where non-respondent households were recorded, and this sheet was submitted daily to the Survey Coordinator

**More on data collection tool**

The questionnaire for data collection in the study was developed using an android application installed in handheld android devices for data collection. The data collected by field investigators each day was uploaded to a secure online server and was made accessible to supervisors and research team members for quality check and analysis. To ensure, the supervisors and the coordinator would daily check the ten percent of the total households interviewed by cross-checking of multiple related questions. The application also had a GPS tracking which mapped the coordinates of houses visited by investigators and enabled to supervisors to revisit random houses for quality check.

**References**

1. Nambiar D, Sankar D H, Negi J, Nair A, Sadanandan R: **Monitoring Universal Health Coverage reforms in primary health care facilities: Creating a framework, selecting and field-testing indicators in Kerala, India**. *PloS one* 2020, **15**(8):e0236169.
