# Supplementary material for: Intersecting sex-related inequalities in self-reported testing for and prevalence of Non-Communicable Disease (NCD) risk factors in Kerala
Source: BMC Public Health. 2022 Mar 19;22:544. doi: 10.1186/s12889-022-12956-w (PMC8933933; doi:10.1186/s12889-022-12956-w)
Supplement: Supplementary file 5 — Additional file 5. Concentration Curves for Blood Pressure and Glucose testing and self-reported prevalence for High Blood Pressure and Glucose by education and wealth. [file 12889_2022_12956_MOESM5_ESM.docx]

**Concentration Curves for Blood Pressure and Glucose testing and self-reported prevalence for High Blood Pressure and Glucose by education and wealth**

**Fig S1: Concentration curves for all four indicators by Education**


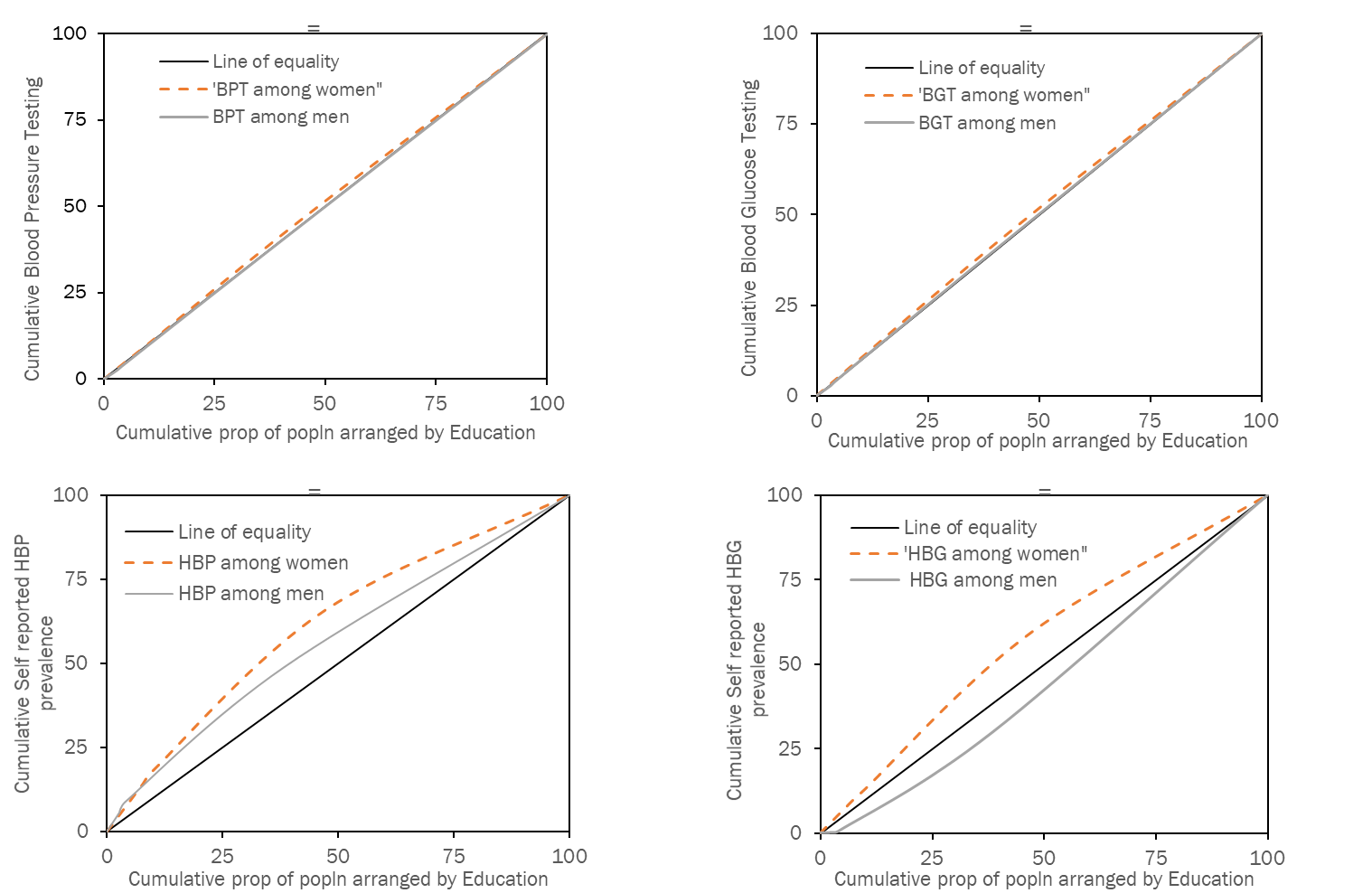


*BPT: Blood Pressure Testing, BGT: Blood Glucose Testing, HBP: High Blood Pressure, HBG: High Blood Glucose

**Fig S2: Concentration curves for all four indicators by Wealth**


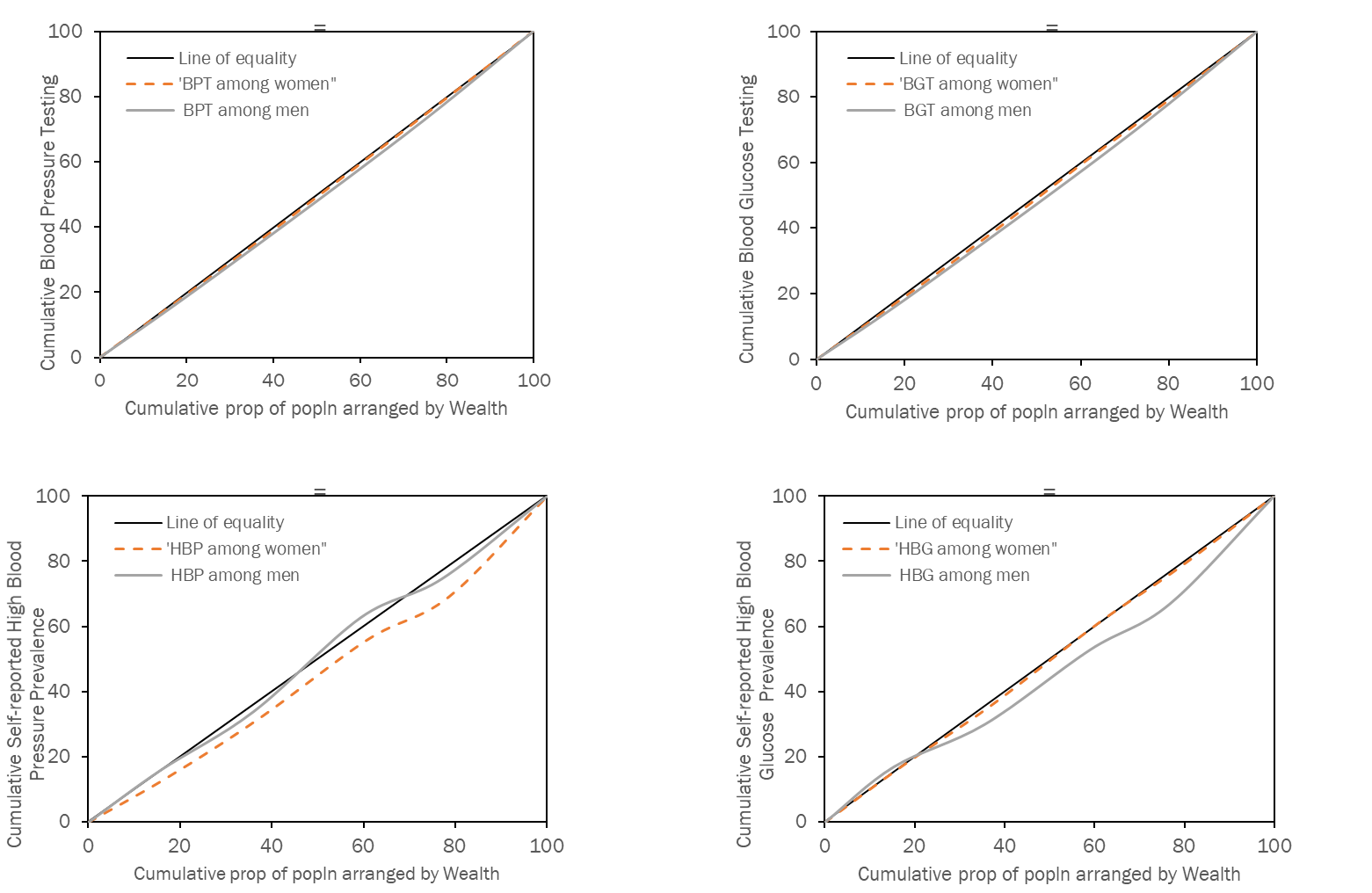


*BPT: Blood Pressure Testing, BGT: Blood Glucose Testing, HBP: High Blood Pressure, HBG: High Blood Glucose
